# Variation in fungal microbiome (mycobiome) and aflatoxin in stored in-shell peanuts at four different areas of China

**DOI:** 10.3389/fmicb.2015.01055

**Published:** 2015-10-22

**Authors:** Ning Ding, Fuguo Xing, Xiao Liu, Jonathan N. Selvaraj, Limin Wang, Yueju Zhao, Yan Wang, Wei Guo, Xiaofeng Dai, Yang Liu

**Affiliations:** Institute of Food Science and Technology – Chinese Academy of Agricultural Sciences/Key Laboratory of Agro-Products Processing, Ministry of AgricultureBeijing, China

**Keywords:** fungal microbiome, mycobiome, AFB_1_, peanuts, storage, ITS sequencing

## Abstract

The contamination of peanuts with *Aspergillus* sp. and subsequently aflatoxins is considered to be one of the most serious safety problems in the world. Mycobiome in peanuts is critical for aflatoxin production and food safety. To evaluate the biodiversity and ecological characteristics of whole communities in stored peanuts, the barcoded Illumina paired-end sequencing of the internal transcribed spacer 2 (ITS2) region of rDNA was used to characterize the peanut mycobiome monthly over a period of 1 year at four main peanut grown areas, i.e., Liaoning (LN, North East), Shandong (SD, East), Hubei (HB, Central), and Guangdong (GD, South) provinces. The fungal diversity of peanuts stored in SD was the highest with 98 OTUs and 43 genera, followed by LN, HB and GD. In peanuts stored in SD, *Rhizopus, Emericella*, and *Clonostachys* were predominant. In peanuts from LN, *Penicillium, Eurotium*, and *Clonostachys* were abundant. In peanuts from HB, *Penicillium, Eurotium*, and *Aspergillus* were higher. In GD peanuts, *Eurotium, Aspergillus*, and *Emericella* were mainly seen. The abundances of *Aspergillus* in LN, SD, HB, and GD were 0.53, 6.29, 10.86, and 25.75%, respectively. From the North of China to the South, that increased over the latitude, suggesting that the higher temperature and relative humidity might increase the risk of peanuts contaminated with *Aspergillus* and aflatoxins. During the storage, *Aspergillus* levels were higher at 7–12 months than in 0–6 months, suggesting that the risk increases over storage time. At 7–10 months, AFB_1_ was higher in four areas, while declined further. The reduction of AFB_1_ might be attributed to the inhibition and degradation of AFB_1_ by *Aspergillus niger* or to the combination with the compounds of peanuts. This is the first study that identified the mycobiome and its variation in stored peanuts using ITS2 sequencing technology, and provides the basis for a detailed characterization of whole mycobiome in peanuts.

## Introduction

Mycotoxins are secondary metabolites produced by different fungal species when they infect the foods and feeds. Mycotoxins contamination is commonly seen in the human food chain, posing great threat to public health and the common mycotoxins are aflatoxin, deoxynivalenol, zearalenone, fumonisins, ochratoxin, patulin, and ergot alkaloids (Hussein and Brasel, 2001). Mycotoxins are considered to be naturally occurring contaminants in peanut, maize, wheat, rice, tree nuts, dried fruit, spices, figs, crude vegetable oils, cottonseed, cocoa beans, copra, and feeds both pre and post-harvest. Mycotoxin producing fungi belong mainly to the genera *Aspergillus, Fusarium, Penicillium*, and *Alternaria* (Gonçalez et al., 2008). Presence of multiple mycotoxins in the same commodity produced by different species is very common.

Aflatoxins are the most toxic and carcinogenic in nature. Aflatoxins have both teratogenic and mutagenic properties and cause toxic hepatitis, hemorrhage, edema, immunosuppression, and hepatic carcinoma (group 1; International Agency for Research on Cancer [Iarc, #186], 1993, 2002), especially aflatoxin B_1_ (AFB_1_). In China, aflatoxins contamination in peanuts and peanut products has been an alarming problem, although they have been found mostly in low level (Ding et al., 2012). In the last decade, more than 28% of food imported from China was rejected by RASFF (Food and Feed Safety Alerts-European Commision) due to aflatoxin contamination^1^.

*Aspergillus flavus* growth and subsequently aflatoxin production in peanuts depend on several factors, such as geographical region, seasonal variation and the environmental factors during planting and storage. Tropical and subtropical regions, with high temperatures and high relative humidity (RH), are highly favorable for *A. flavus* growth and aflatoxin production (Smith and Ross, 1991; Giorni et al., 2009). In addition, some filamentous fungi also could influence *A. flavus* growth and aflatoxin accumulation. For example, *Aspergillus niger* significantly suppressed *A. flavus* growth, sporulation and reduced the level of AFB_1_ (Krishnamurthy and Shashikala, 2006; Cvetnić and Pepeljnjak, 2007; Xu et al., 2013). *Aspergillus oryzae* inhibited AFB_1_ biosynthesis during the fermentation of soy sauce (Maing et al., 1973); *Aspergillus chevalieri* and *Aspergillus candidus* reduced AFB_1_ production by *Aspergillus parasiticus* in rice (Boller and Schroeder, 1973, 1974); and *Alternaria* sp., *Cladosporium* sp., and *Mucor* sp. decreased AFB_1_ production by *A. flavus* NRRL 3251 (Cvetnić and Pepeljnjak, 2007). Therefore, it is necessary to characterize the fungal microbiome of peanuts and its variations during storage. Nakai et al. (2008) evaluated the mycoflora and occurrence of aflatoxins in stored peanut samples from Brazil and found that *Fusarium* sp. and *Aspergillus* sp. were highly predominance with the presence of five other genera. However, the approach used by this group provided only a limited snap shot of the fungal members of the microbiome. In recent years, high-throughput sequencing technologies have opened new frontiers in microbial community analyses by providing an economic and efficient means to identify the microbial phylotypes in samples. Studies have revolutionized our understanding of the microbial communities present in our human bodies (Costello et al., 2009; Grice et al., 2009; Ghannoum et al., 2010), soils (Roesch et al., 2007; Lauber et al., 2009) and deep seas (Sogin et al., 2006). The internal transcribed spacer 2 (ITS2), which is an excellent phylogenetic marker suitable for fungal taxon assignment (Mello et al., 2011), has been successfully used in comparative ecology studies where it gives results that are convergent with, if not comparable to, those for other markers (Mello et al., 2011; Arfi et al., 2012). ITS-based surveys are extremely valuable because they allow the assessment of biodiversity and ecological characteristics of whole communities or individual microbial taxa.

Although some previous studies have evaluated the mycoflora in stored peanuts using traditional isolation, enumeration and identification, the approach used by these researches provided only a limited snap shot of the fungal members of the microbiome. To obtain a more comprehensive profile of the fungal microbiome (mycobiome), we used the barcoded Illumina paired-end sequencing (BIPES) method (Zhou et al., 2011) in this study to characterize the mycobiome and its variation in stored peanuts at four different areas, Liaoning, Shandong, Hubei, and Guangdong. This is the first study that characterizes the mycobiome and its variation in stored peanuts using ITS2 sequencing. The findings of this study are likely to encourage the implementation and design of mold and aflatoxin management strategies.

## Materials and Methods

### Ethics Statement

Specific permission was not needed for our field studies. The peanuts variety used in our field study was main cultivar in Liaoning (LN), Shandong (SD), Hubei (HB), and Guangdong (GD) province. No transgenic or mutant plant has been used in our study. Also we confirm that the field studies did not involve endangered or protected species.

### Sample Preparation

Peanuts (Virginia type) were obtained from four major peanut production areas in China, which are Jinzhou city of Liaoning province (North East), Linyi city of Shandong province (East), Xiangyang city of Hubei province (Central), and Zhanjiang city of Guangdong province (South), located in the Northeast plain, Yellow River valley, the center of Yangtze River valley and the Southeast Coastal areas, respectively. After harvest and drying, the peanuts were placed in 25 kg bags (a total of three bags each area), piled up on a wooden frame, and stored for a period of 12 months in a warehouse with ventilation 14 h each day (8:00–22:00). Before storage, the warehouse was sterilized with 30 mg/m^3^ ozone. Samples were removed monthly from different points of each bag until completing a sample of 1 kg (Nakai et al., 2008). Three samples (triplicates) were collected each month (0–12 months) in each area, for a total 156 samples (three samples/area/month × 4 area × 13 months = 156 samples), and were mailed in hermetic bags to our lab in Beijing. Each sample was analyzed for aflatoxins in peanut kernels. Three samples of one area each month were mixed, thus 52 samples were analyzed for mycobiome. The water activities of peanut kernels were determined by using Aqualab Series 4 (Decagon Devices, Inc., Pullman, WA, USA).

### Total Microbiome Genomic DNA Extraction

One hundred grams of peanut kernels from the collected samples washed in 100 ml sterile water and the water samples were collected and vacuum-filtered through 0.22 μm filters within. The filters containing sample were placed in 50 ml tubes and stored at -20°C until analysis. The MoBio Power Water^®^ DNA Isolation Kit (MoBio Laboratories, Inc., Carlsbad, CA, USA) was used to extract the genomic DNA from the filters according to manufacturer’s instruction. The final DNA elution was performed using sterile deionized water. DNA quality and quantity were measured by spectrophotometric quantification in a NanoDrop 1000 (Thermo Fisher Scientific, Inc., Newark, DE, USA) and by agarose gel electrophoresis. Extracted DNA was stored at -80°C.

### PCR Amplification and ITS2 Sequencing

Fungal universal primers ITS2F (5′-GCATCGATGAAGAACGCAGC-3′) and ITS2R (5′-TCCTCCGCTTATTGATATGC-3′) were used to amplify the ITS2 region of fungal rDNA. PCR reactions were carried out in a total reaction volume of 30 μl consisting of 15 μl of Phusion High-Fidelity PCR Master Mix (New England Biolabs, Inc., Ipswich, MA, USA), 0.2 μM of forward and reverse primers, and 10 ng of template DNA. The PCR amplification program consisted of a initial heating to 98°C for 1 min, 30 cycles of denaturation at 98°C for 10 s, annealing at 50°C for 30 s and extension at 72°C for 60 s, followed by a 5-min extension at 72°C. GeneJET Gel Extraction Kit (Thermo Scientific, South Logan, UT 84321, USA) was used to purify the amplified products according to the manufacturer’s recommendations. Of 52 peanut samples, 39 samples were successfully amplified. Amplicons were quantified and sequencing libraries were generated using NEB Next Ultra DNA library Prep Kit for Illumina (New England Biolabs, Inc., USA) according to manufacturer’s instructions. The amplicon libraries were subsequently sequenced on an Illumina MiSeq platform at Novogene (Novogene, Beijing, China).

### Bioinformatics Analyses

FLASH (Magoc and Salzberg, 2011) was used to merge the paired-end reads from the original DNA fragments. QIIME software package^2^ and UPARSE pipeline were used to analyze the reads and pick operational taxonomic units (OTUs; Gillever et al., 2010). Sequences were assigned to OTUs at 97% similarity. A representative sequence for each OTU was picked and used the RDP classifier (Wang et al., 2007) to assign taxonomic data to each representative sequence. In order to reveal Alpha diversity, rarefaction curves were generated based on these two metric: the observed species metric is simply the count of unique OTUs found in the sample, and Shannon index.

### Determination of Aflatoxins

Aflatoxins in the peanuts were detected by the HPLC (high performance liquid chromatography). The specific methodology and technology were refer to Chinese standard methods (Liu et al., 2006) and AOAC method 994.08 (AOAC AOAC International, 2000) with minor modifications. In this research, 50 g peanut kernels were mixed, kept in hermetic bags, and stored at -20°C until analysis. Peanut kernels (5 g) were extracted with 15 ml of acetonitrile:water (84:16, v/v). After ultrasonic extraction (50°C, 10 min) and filtration with double-layer slow quantitative filter paper, 4 ml of the resulting filtrate was collected in 10 ml test tube, and then mixed with 2 ml petroleum ether. The mixture was blended using vortex control for 30 s and stood for 15–20 min to separate into two layers. The lower solution (3 ml) was collected, and mixed with 8 ml pure water and further filtered through a 0.45 μm organic membrane. Extracts (8 ml) were passed through immunoaffinity columns (ToxinFast Columns, Cat. No. HCM0125, HuaanMagnech Bio-tech, Beijing, China) with a flow rate of one droplet per second and eluted using 2 ml of methanol into glass tubes. The eluate was evaporated to dryness under a stream of nitrogen gas at 60°C. The purified extract was re-dissolved with 1 ml of acetonitrile: water (15: 85, v/v) and quantified by HPLC-FD. This method is applicable for determination of afltoxins B_1_, B_2_, G_1_, and G_2_ at ≥1 ng total aflatoxins/g in raw peanuts.

High performance liquid chromatography analysis was performed by using Waters 2695 (Waters Corporation, Milford, MA, USA) coupled to a Waters 2475 fluorescence detector (λexc 360 nm; λem 440 nm) and a post-column derivation system, and an Agilent TC-C18 column (250 mm × 4.6 mm, 5 μm particle size). The mobile phase (water:methanol:acetonitrile, 4:1:1) was pumped at a flow rate of 0.5 ml/min. AFB_1_, B_2_, G_1_, and G_2_ (Sigma-Aldrich, St. Louis, MO, USA) were used as standards. The calibration curves were set up for each type of aflatoxin by the external standard method with five standard concentrations: AFB_1_: 1.0, 2.0, 5.0, 10.0, and 20.0 ng/ml (*r*^2^ = 0.998); AFB_2_: 1.0, 1.5, 2.0, 2.5, and 4.0 ng/ml (*r*^2^ = 0.999); AFG_1_: 2.5, 3.5, 5.0, 7.0, and 10.0 ng/ml (*r*^2^ = 0.996); AFG_2_: 2.0, 2.5, 3.5, 5.0, 7.5 ng/ml (*r*^2^ = 0.999). The HPLC quantification and detection limits were: 0.5 and 0.1 ng/mL for AFB_1_; 0.1 and 0.05 ng/mL for AFB_2_; 2.0 and 1.0 ng/mL for AFG_1_ and 0.15 and 0.05 ng/mL for AFG_2_. Recoveries for standard AFB_1_, B_2_, G_1_, and G_2_ were 85–95, 84–96, 89–95, 76–85%, respectively. The repeatability (RSDr, *n* = 6) ranged from 6.2 to 11.2% for AFB_1_, from 6.1 to 10.5% for AFB_2_, from 6.3 to 14.8% for AFG_1_, and from 6.3 to 14.3% for AFG_2_. The reproducibility (RSDR, *n* = 10) ranged from 7.1 to 14.3% for AFB_1_, 7.2 to 14.4% for AFB_2_, 11.8 to 18.3% for AFG_1_, and 7.2 to 22.6% for AFG_2_.

### Isolation and Identification of *Aspergillus* Fungi from Peanut Kernels

Approximately 30 g was removed from each of the samples and disinfected with 0.4% sodium hypochlorite solution for 2 min, followed by washing with sterile distilled water for elimination of external contaminants (Berjak, 1984; Nakai et al., 2008). The first isolation was performed using Dichloran Rose Bengal Chloramphenicol agar media (DRBC; Pitt et al., 1993). Three plates containing 11 kernels were used for each sample. The plates were incubated at 28°C for 5 days. The colonies belonging to the genus *Aspergillus* were identified to the species level according to Pitt and Hocking (1997). To verify the results of morphological characterization and identification of fungi, 18S rDNA was amplified by PCR and sequenced. The PCR primers were ITS1 (TCCGTAGGTGAACCTGCGG) and ITS4 (TCCTCCGCTTATTGATATGC). PCR conditions were as follows: 95°C for 3 min, 35 cycles at 95°C for 30 s, 55°C for 30 s and 72°C for 1 min. Basic Local Alignment Search Tool (BLAST) was used to identify the closest affiliated sequence in the GenBank/NCBI dataset. To confirm the identification of *A. flavus* and *A. paraciticus*, these isolates were transferred to *A. flavus* and *A. parasiticus* Agar (AFPA) and incubated for 7 days unilluminated at 31°C. On this medium *A. flavus* and *A. parasiticus* produce a bright orange reverse (Pitt et al., 1983).

### Degradation of AFB_1_ in PDB Medium by *Aspergillus niger*

Twenty strains of *A. niger* were previously confirmed by biosynthesis and HPLC as non-producer of AFB_1_. One ml of *A. niger* conidia suspension (of 10^6^ conidia/ml) was inoculated in 50 ml of PDB (patato 200 g/l, glucose 20 g/l) with 100 ng/ml of AFB_1_. Flasks were incubated at 28°C for 5 days under agitation in a ZWY-2102C incubator (Zhicheng, Shanghai, China). All treatments were tested in triplicate.

AFB_1_ was isolated and detected using HPLC. Briefly, 10 ml of liquid medium was extracted with 10 ml of chloroform, the extract was evaporated in N_2_ flow, and the residue was dissolved in 2 ml of acetonitrile. The extracts were cleaned using an immunoaffinity column (ToxinFast Columns). The determination of AFB_1_ levels were performed by HPLC.

### Degradation of AFB_1_ in Peanuts by *Aspergillus niger*

One ml of *A. niger* conidia suspension (of 10^6^ conidia/ml) was inoculated on the 50 g peanuts (*a*_w_ 0.90) with 62.3 μg/kg of AFB_1_. The peanut samples were incubated at 28°C for 5 days in an incubator. All treatments were performed in triplicate. AFB_1_ in peanuts was isolated and detected by the HPLC according to Section “Determination of Aflatoxins.”

### Climatic Data

**FIGURE 1:**
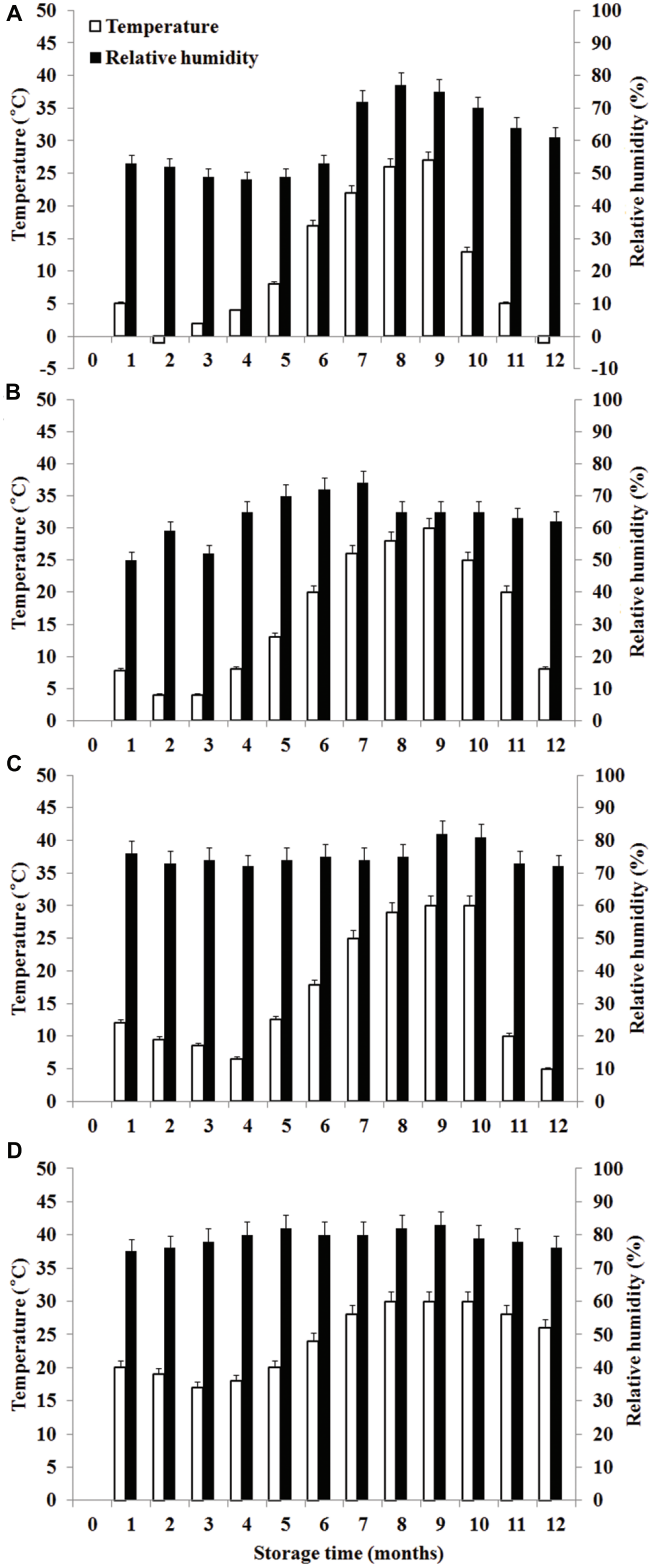
**Temperature and relative humidity in four storage areas. (A)** Liaoning, **(B)** Shandong, **(C)** Hubei, **(D)** Guangdong.

During the storage period, climatic data such as temperature (°C) and RH were recorded monthly in the regions of the experiment. Daily mean temperature is the average value of temperature at 2:00, 8:00, 14:00, and 20:00 each day. Monthly mean temperature is the average value of daily mean temperature in 1 month. The calculation of daily mean RH and monthly mean RH is similar with temperature. The temperature and RH in four storage areas were shown in **Figure 1**.

### Disposal of Experimental Material Contaminated with Aflatoxins

All the peanuts and experimental material contaminated with aflatoxins were incinerated in a high-temperature incinerator (Incinerator ZDEK, CECO Corporation, Tianjin, China) at 600°C for 2 h. Later the materials were discarded in special bags for further disposal.

### Statistical Analyses

All the experiments results were evaluated using analysis of variance (ANOVA) for multiple comparisons followed by the Turkey test. Differences were considered significant at *p* < 0.05.

## Results

### Data Characteristics

Of 52 peanut samples stored in four areas of China, 39 samples were successfully amplified and sequenced. DNA from the peanut samples stored from 0 to 3 months in LN and SD province and 0 to 2 months in HB and GD province could not be amplified. The results suggested that fungi number is lesser in early stages of storage. The results were consistent with the findings of other investigators studying peanuts from Brazil (Rossetto et al., 2005; Nakai et al., 2008) and India (Bhattacharya and Raha, 2002) using traditional approach. They found that the frequencies of fungi, especially *Aspergillus* sp., were higher in peanut kernels in later stages of storage. In peanut kernels stored in LN, the average number of raw reads generated per sample was 85,099, of which 74,457 were retained following filtering and denoising steps, and 68,699 reads were subsequently clustered into different OTUs. The number of reads which were clustered into OTUs in LN was highest, followed by SD, GD, and HB (**Table 1**).

**Table 1:** Summary of pyrosequencing analysis.

Storage	Liaoning		Shandong		Hubei		Guangdong	
Time (month)	Number of reads	Average read length (bp)	Number of reads	Average read length (bp)	Number of reads	Average read length (bp)	Number of reads	Average read length (bp)
0								
01								
02								
03					13332	327	27894	315
04	109021	312	54768	319	59821	305	70874	322
05	68360	314	36687	324	60619	305	36187	316
06	105223	307	142626	322	56238	307	81927	337
07	76784	310	32989	318	41767	309	85138	315
08	38472	337	60854	316	38531	305	35774	321
09	53805	314	54066	322	32518	305	26291	348
10	59344	310	46869	344	35420	315	22819	352
11	47366	321	67531	356	56711	326	82132	317
12	59644	312	59294	358	48809	326	65899	323
**Average**	68669	315	61743	331	44377	313	53494	327

### Fungal Diversity in Peanut Kernels in Different Areas

As shown in **Table 2**, in peanuts stored in LN, HB and GD, Ascomycota was dominant, 93.60%, 92.27% and 86.49% of reads belongs to the phylum, respectively. Among Ascomycota, *Penicillium, Eurotium, Aspergillus, Emericella, Talaromyces, Clonostachys, Cladosporium*, and *Alternaria* were main genera observed. However, in peanut kernels stored in SD, the dominant phyla were Ascomycota and Zygomycota. *Rhizopus, Emericella, Clonostachys*, and *Aspergillus* were predominant genera. The predominant species in *Aspergillus* were *A. flavus* and *A. penicillioides*. In peanuts stored in LN, SD, HB and GD, the average percent of reads of two species were 0.12 and 2.01%, 6.39 and 0.26%, 1.41 and 10.33%, 0.22 and 31.59%, respectively. In *Penicillium, P. georgiense, Penicillium* sp. *ASR_162, Penicillium* sp. *III JH_2010* and *Penicillium* sp. *PSF11* were main fungi species. In *Emericella, Talaromyces, Cladosporium, Macrophomina, Clonostachys*, and *Rhizopus* genera, the main species were *Emericella nidulans, Talaromyces* sp. *NRRL 62223, Cladosporium* sp. *4 MU_202, Macrophomina phaseolina, Clonostachys rosea*, and *Rhizopus arrhizus*, respectively.

**Table 2:** Distribution of operational taxonomic units (OTUs) among fungal lineages including the 10 most abundant genera detected in stored peanuts of four storage areas.

Taxnomic affinity	Percent of reads			
	Liaoning	Shandong	Hubei	Guangdong
Ascomycota	93.60	72.90	92.27	86.49
Eurotiomycetes	70.34	30.68	86.22	85.09
*Eurotium*	16.14	0.96	14.59	50.04
*Aspergillus*	0.53	6.29	10.86	25.75
*Penicillium*	43.86	0.79	55.51	2.69
*Emericella*	5.17	22.46	4.32	6.55
*Talaromyces*	3.87	0.08	0.87	0.01
Dothideomycetes	5.66	4.18	1.62	0.11
*Alternaria*	1.29	0.81	0.53	0.01
*Cladosporium*	2.72	2.26	0.39	0.06
Sordariomycetes	17.24	37.24	1.53	0.32
*Clonostachys*	15.12	19.90	0.43	0.18
*Nectriaceae* sp.	0.22	7.67	0.34	0.04
Zygomycota	0.19	26.22	0.78	0.16
Mucomycotina	0.18	26.21	0.77	0.16
*Rhizopus*	0.18	26.21	0.77	0.16

### Storage Time and Fungal Diversity

**FIGURE 2:**
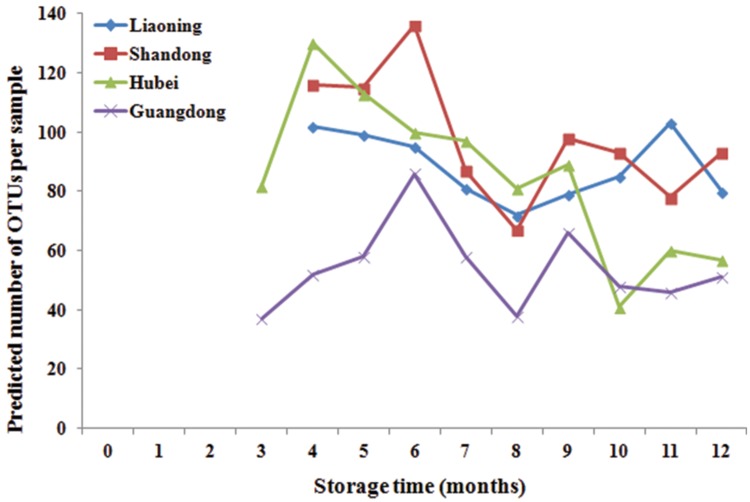
**Predicted number of operational taxonomic units (OTUs) per sample in stored peanuts of four storage areas**.

Significant variation in per-sample OTU richness based on storage time and peanuts varieties stored in different areas was observed (**Figure 2**). The OTUs number in peanuts stored in SD was the highest, followed by LN, HB, and GD. The average number of OTUs per sample in peanuts stored in SD was 98, reaching its highest and lowest value at 6th and 8th months, respectively. The average numbers of OTUs in LN, HB, and GD have the similar trend. These results suggested that the fungi diversities were fluctuated during the storage. In general the fungi diversity was lowest at the 8th months, when the temperature and RH were high and suitable for the growth of Ascomycota fungi (**Figure 1**).

### Fungal Community Variation in Genus Level Across Storage Time

**FIGURE 3:**
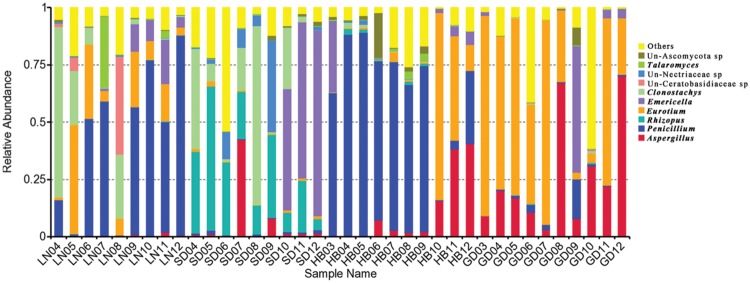
**Overall distribution of fungi at genus level in stored peanuts of four storage areas**.

**FIGURE 4:**
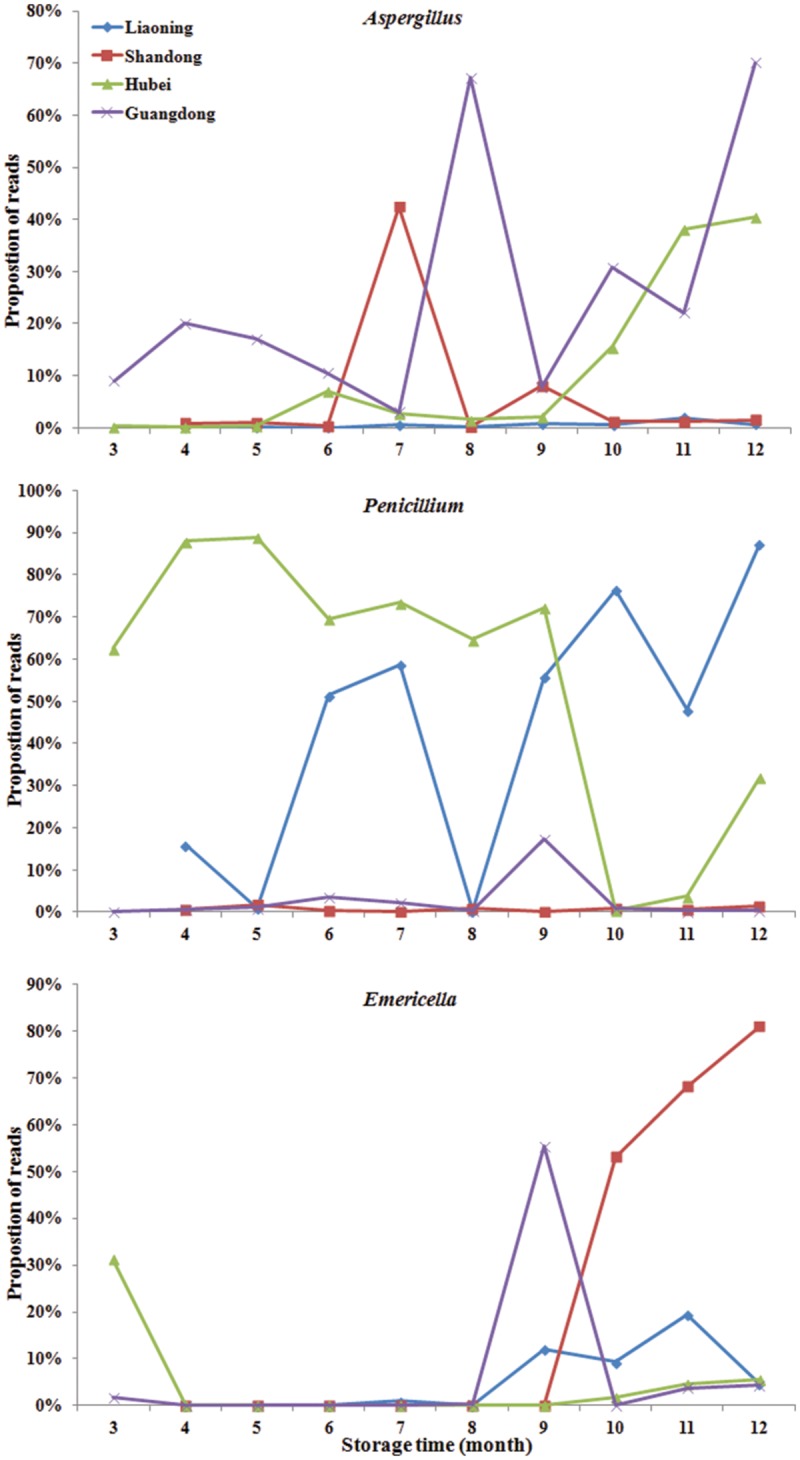
**The relative abundance of predominant taxonomic groups at genus level over storage time in stored peanuts. (A)** *Aspergillus*, **(B)** *Penicillium*, **(C)** *Emericella*.

Significant variation in the relative abundance of fungal genera per sample based on storage areas and storage time was observed (**Figures 3** and **4**). In peanuts of LN, *Aspergillus* sp. were rare and only had a relative abundance of 0.53%. The relative abundances of *Aspergillus* sp. at 9th to 12th months were higher than that at 4th to 8th months. *Aspergillus* sp. exhibited a peak in relative abundance at 11th months, while this peak occurred at 7th, 12th and 12th, in SD, HB, and GD, respectively. In general, the abundances of *Aspergillus* sp. were higher in later stage of storage than the early stage. The relative abundances of *Penicillium* sp. in LN was higher than 50% at the 6th, 7th, 9th, 10th, and 12th months, and exhibited a peak at 12th month, while this peak occurred at 5th, 5th and 9th, in SD, HB, and GD, respectively. In LN and GD, the relative abundances of *Emericella* sp. were higher at 9th to 12th months than the early stages of storage. And in SD and HB, *Emericella* sp. sharply increased at 10th month and were higher at 10th to 12th months than other months. These results suggested that the relative abundances of *Aspergillus* sp. and *Emericella* sp. increased in the later stage of storage and higher than the early stage of storage.

### Isolation and Identification of *Aspergillus* Fungi from Peanut Kernels

In the peanut samples the following mycotbiota were isolated: *Aspergillus* sp. *Penicillium* sp. *Rhizoupus* sp. and *Fusarium* sp. Of the *Aspergillus, A. flavus* was the most important because of its known toxigenic potential. In the present study, 100 isolates of *Aspergillus* sp. were sequenced and identified, and *A. flavus, A. penicillioides*, and *A. niger* were identified. Of them, the percentage of *A. penicillioides* (60%) was the highest, followed by *A. flavus* (21%) and *A. niger* (17%). On AFPA medium, 21 isolates produced a bright orange reverse and were confirmed to be *A. flavus.*

### Aflatoxin B_1_ in Stored Peanuts

AFB_2_, AFG_1_, and AFG_2_ were not detected in all the peanut samples. The variation of AFB_1_ in peanuts stored in four areas was shown in **Table 3**. During the first 6 months of the storage, AFB_1_ was not detected in peanuts of four areas. AFB_1_ was detected in peanuts of four areas at the 7th month, then increased and reached the highest level at the 8th or 9th month. Subsequently, AFB_1_ levels decreased and were not detectable at 11th and 12th months in peanuts of all areas.

**Table 3:** Mean levels of aflatoxin B_1_ in stored peanuts.

Storage time (month)	Liaoning	Shandong	Hubei	Guangdong
0	ND	ND	ND	ND
1	ND	ND	ND	ND
2	ND	ND	ND	ND
3	ND	ND	ND	ND
4	ND	ND	ND	ND
5	ND	ND	ND	ND
6	ND	ND	ND	ND
7	2.63 ± 0.13	3.25 ± 0.12	1.48 ± 0.28	2.29 ± 0.15
8	2.87 ± 0.15	3.52 ± 0.18	1.51 ± 0.09	3.65 ± 0.21
9	2.65 ± 0.20	3.61 ± 0.14	1.59 ± 0.10	4.03 ± 0.11
10	1.02 ± 0.21	2.18 ± 0.18	ND	1.27 ± 0.32
11	ND	ND	ND	ND
12	ND	ND	ND	ND

*ND, not detected.*

### Degradation of AFB_1_ by the Culture Filtrate of *A. niger*

The culture of 20 strains of *A. niger*, isolated from the stored peanuts, all could significantly degrade AFB_1_ with degradation rate of 44.5–100% (**Table 4**). Of 20 strains, eight strains of *A. niger* could completely degrade AFB_1_. Furthermore, *A. niger* N-01, N-02, and N-03 reduced AFB_1_ in peanuts from 62.3 ± 4.5 μg/kg to undetectable with degradation rate of 100%. The results suggested *A. niger* strains in stored peanuts might have the ability to reduce AFB_1_ levels in peanuts.

**Table 4:** The degradation of AFB_1_ by *Aspergillus niger.*

Identification number	In PDB		In kernels	
	AFB_1_ (μg/kg)	Degradation rate (%)	AFB_1_ (μg/kg)	Degradation rate (%)
Control	80.2 ± 5.5	0	62.3 ± 4.5	0
N-01	0	100	0	100
N-02	0	100	0	100
N-03	0	100	0	100
N-04	10.0 ± 4.2^∗∗^	90.0	14.3 ± 3.5^∗∗^	77.0
N-05	21.4 ± 7.5^∗∗^	78.6	36.5 ± 4.3^∗^	41.4
N-06	0	100	8.5 ± 1.4^∗∗^	86.4
N-07	6.4 ± 3.1^∗∗^	93.6	11.8 ± 2.6^∗∗^	81.1
N-08	0	0	9.1 ± 1.3^∗∗^	85.4
N-09	11.0 ± 1.4^∗∗^	89.0	16.9 ± 4.2^∗∗^	72.9
N-10	20.1 ± 1.9^∗∗^	79.9	40.6 ± 6.4^∗^	34.8
N-11	0	100	12.5 ± 3.1^∗∗^	79.9
N-12	10.7 ± 1.9^∗∗^	89.3	16.4 ± 4.3^∗∗^	73.7
N-13	13.0 ± 0.5^∗∗^	87.0	18.2 ± 5.4^∗∗^	70.8
N-14	0	100	10.5 ± 1.7^∗∗^	83.1
N-15	0	100	13.6 ± 1.8^∗∗^	78.2
N-16	12.4 ± 1.4^∗∗^	87.6	18.7 ± 4.1^∗∗^	70.0
N-17	0	100	13.5 ± 3.4^∗∗^	78.3
N-18	10.0 ± 1.9^∗∗^	90.0	15.8 ± 3.5^∗∗^	74.6
N-19	14.0 ± 1.9^∗∗^	86.0	24.5 ± 3.1^∗∗^	60.7
N-20	55.5 ± 8.5^∗∗^	44.5	60.4 ± 8.7	3.0

*^∗^Statistically significant when compared to Control, *P* < 0.05; ^∗∗^Statistically significant when compared to Control, *P* < 0.01.*

## Discussion

The average numbers of OTUs in peanuts of LN, SD, HB, and GD were 88, 98, 85, and 56, respectively, with 33, 43, 35, and 22 fungal genera being identified, respectively. These results showed that the fungal diversity of peanuts stored in SD was the highest, and suggested that the moderate climate of SD was more suitable for maintaining fungal diversity (**Figure 1**). However, the fungal diversity of peanuts stored in GD was lowest because the temperature and RH were greatly higher than other areas (**Figure 1**). Of 22 genera, *Eurotium, Aspergillus, Emericella*, and *Penicillium* were the predominant genera, total relative abundance of the four genera was more than 85%.

In general, *Eurotium, Penicillium, Aspergillus, Clonostachys, Emericella*, and *Rhizopus* were main genera in peanuts stored in four areas, because of the greater adaptation of these fungi to the substrate, especially during storage (Rossetto et al., 2005; Nakai et al., 2008). The occurrence of *Aspergillus, Penicillium*, and *Rhizopus* was similar to the findings of other investigators studying peanuts from Brazil (Rossetto et al., 2005; Nakai et al., 2008) and India (Bhattacharya and Raha, 2002) using traditional isolation, enumeration and identification methods of the mycoflora on DRBC and AFPA media. *Eurotium, Clonostaschys*, and *Emericella* were not detected in these studies as they did not grow well on DRBC or AFPA media.

During the storage, in general the relative abundances of *Aspergillus* were higher at 7th to 12th months than the first 6 months. The highest value in LN, SD, HB, and GD were observed at the 11th, 7th, 12th, and 12th months, respectively. In particular, the relative abundances of peanuts stored in HB sharply increased from 9 to 12 months reaching the highest value 40.40%. The result was consistent with the findings of other investigators studying peanuts from Brazil (Rossetto et al., 2005; Nakai et al., 2008) and India (Bhattacharya and Raha, 2002) using traditional approaches. They found that the frequencies of *Aspergillus* sp. were higher in peanut kernels at 7th to 12th months than the first 6 months. On the other hand, the increase in the frequency of *Aspergillus* sp. can be explained by the fact of that this fungus and *Penicillium* sp. are considered to be storage fungi (Nakai et al., 2008; Krijgsheld et al., 2012). These results confirmed that the risks of peanuts contaminated with aflatoxins increased over storage time, especially after 6 months.

**FIGURE 5:**
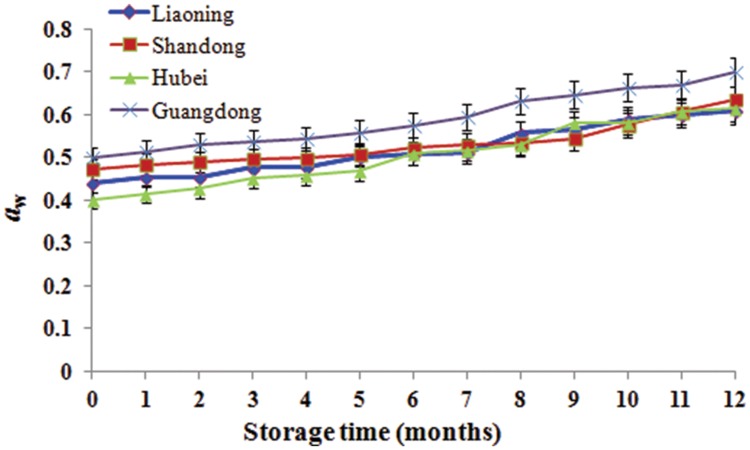
**The water activity (*a*_w_) of stored peanuts over storage time**.

During storage from 7th to 10th months, AFB_1_ increased and was high because it was summer with the highest temperature and RH. However, AFB_1_ concentrations were lower than 5 μg/kg during storage. Studies conducted in China analyzing the occurrence of aflatoxins in peanuts and derived products generally reported similar contamination levels (Ding et al., 2012). Furthermore, the relative abundances of *A. flavus* were low (0.06–6.04%). The result mainly be attributed to low *a*_w_ (0.40–0.70; **Figure 5**) which was below the minimum range of 0.70–0.80 established for the growth of *A. flavus* (Hocking, 1997) and the minimum range of 0.80–0.90 for aflatoxins production (Passone et al., 2010, 2012). Furthermore, the mean temperature ranged from -1 to 30°C and the RH ranged from 48 to 83% (**Figure 1**). Thus, the temperature was below 32–33°C considered to be the optimum temperature for the growth of *A. flavus* by Hocking (1997), the RH was lower than 83–85% considered to favor the growth of *A. flavus* (Christensen et al., 1977). Kaaya and Kyamuhangire (2006) obtained the similar results. They found that aflatoxin levels of maize kernels stored for more than 6 months were higher than their counterparts stored for 2–6 months. However, Nakai et al. (2008) found the absence of significant correlations between the presence of AFB_1_ and AFB_2_ in peanut kernels, storage time and abiotic factors.

At 9th to 12th months, the relative abundances of *Aspergillus* sp. increased over storage time, especially in peanuts of HB and GD. However, AFB_1_ concentration of peanuts decreased over times during the same period. So, it was postulated that AFB_1_ reduction was due to degradation by other fungi or binding with other compounds of peanuts. Cvetnić and Pepeljnjak (2007) showed that some mold species such as *Alternaria* sp., *Cladosporium* sp., *Mucor* sp. and *A. niger*, could effectively inhibit the mycelia growth of *A. flavus* and reduce the AFB_1_ production with co-culturing in yeast extract sucrose (YES) broth. In particular, the interaction of *A. niger* with *A. flavus* against production of AFB_1_ has been reported. Nevertheless, the mechanism of AFB_1_ reduction by *A. niger* was still debatable. Horn and Wicklow (1983) indicated that *A. niger* lowered substrate to acidic pH value to suppress AFB_1_ formation. Shantha et al. (1990) demonstrated that oxalic acid and gluconic acid secreted by *A. niger* could prevent AFB_1_ production (Shantha and Rati, 1990; Shantha et al., 1990). However, some recent researches indicated that an antifungal peptide (AFP) isolated from *A. niger* could inhibit the growth of *A. flavus* and AFB_1_ biosynthesis (Lee et al., 1999; Xu et al., 2013). In addition, Xu et al. (2013) in their studies also found that the culture filtrate of strain FS10 could effectively degrade AFB_1_. Interestingly, in the present study, we also found 20 strains of *A. niger* from stored peanuts could degrade AFB_1_. Furthermore, *A. niger* N-01, N-02, and N-03 could reduce AFB_1_ in peanuts.

The present study is the first study that provides a more comprehensive profile of the mycobiome and its variation in stored peanuts using ITS2 sequencing technology. The fungal diversity of peanuts stored in SD was the highest with 98 OTUs and 43 genera, followed by LN, HB, and GD. In peanuts, *Aspergillus, Rhizopus, Emericella, Penicillium, Eurotium*, and *Clonostachys* were predominant. The abundance of *Aspergillus* increased over the latitude (from LN to GD), confirming that the higher temperature and RH may result in the higher risk of peanuts contaminated with *Aspergillus* and aflatoxins. During the storage, *Aspergillus* levels at 7–12 months were higher than first 6 months, suggesting the risk will increase over storage time. During storage 7–10 months, AFB_1_ increased and was high in four areas, and then decreased. The reduction of AFB_1_ might be attributed to the degradation of AFB_1_ by *A. niger* or binding with other compounds of peanuts.

## Author Contributions

YL, XD and FX designed the experiments; ND, FX, XL, JNS, LW, YZ, YW, performed the experiments; FX analyzed the sequencing data; ND analyzed the aflatoxins determination data; XL analyzed the AFB1 degradation data; WG provided scientific expertise; FX wrote the manuscript; YL and JNS edited the manuscript.

## Conflict of Interest Statement

The authors declare that the research was conducted in the absence of any commercial or financial relationships that could be construed as a potential conflict of interest.

## Footnotes

1 https://webgate.ec.europa.eu/rasff-window/portal/?event=searchForm&cleanSearch=1

2 http://qiime.org/

## Supplementary Material

Figure S1 **Agrose electrophoresis of total microbiome genomic DNA extraction from stored peanut kernels**.
