# Supplementary figures and images for: Variation in fungal microbiome (mycobiome) and aflatoxin in stored in-shell peanuts at four different areas of China
Source: Front Microbiol. 2015 Oct 22;6:1055. doi: 10.3389/fmicb.2015.01055 (PMC4614231; doi:10.3389/fmicb.2015.01055)

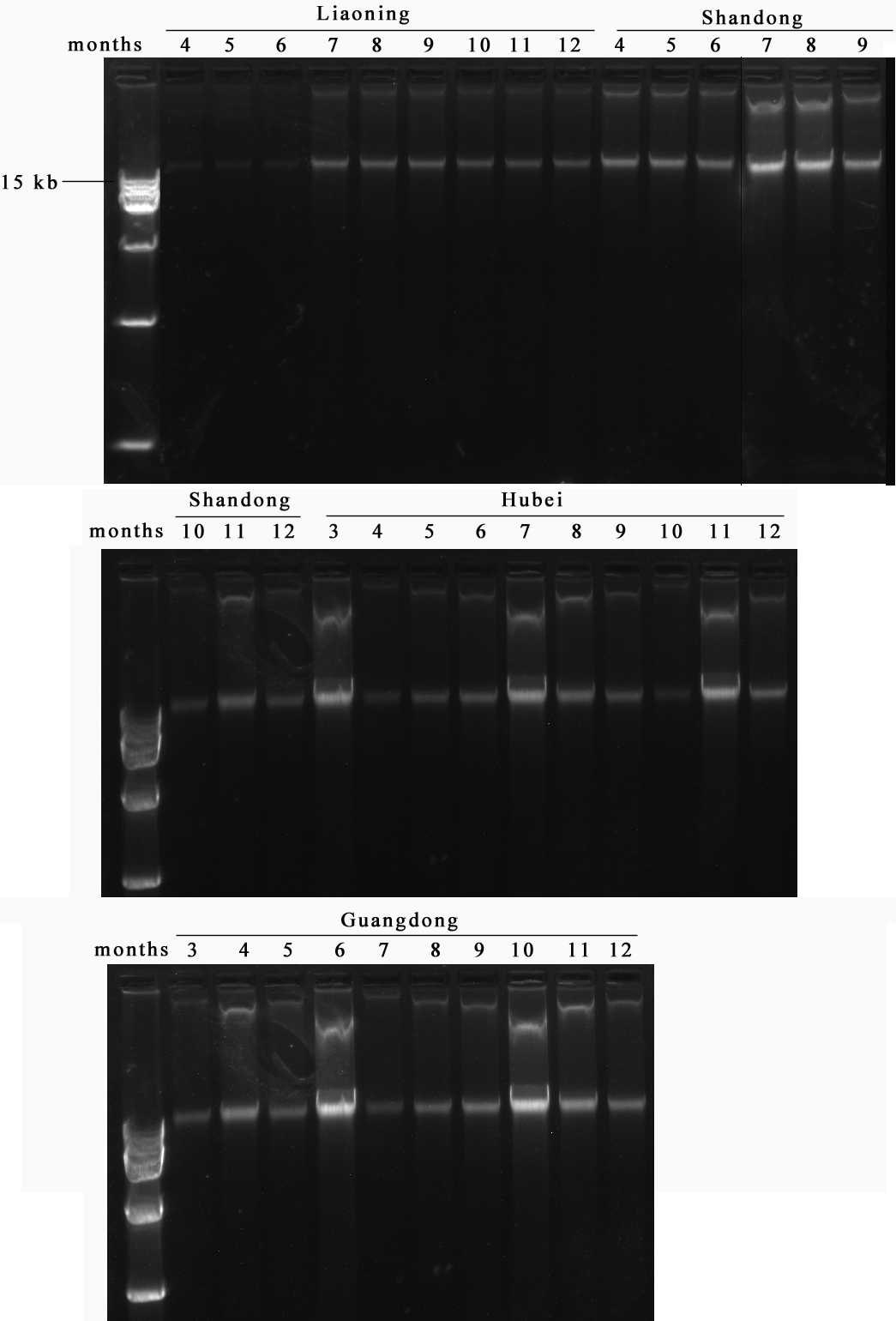

Supplement: Figure S1 — Agrose electrophoresis of total microbiome genomic DNA extraction from stored peanut kernels. [file Image_1.TIF]
